# Decrease of free radical concentrations in humans following consumption of a high antioxidant capacity natural product

**DOI:** 10.1002/fsn3.146

**Published:** 2014-07-18

**Authors:** Boris Nemzer, Tony Chang, Zhuohong Xie, Zbigniew Pietrzkowski, Tania Reyes, Boxin Ou

**Affiliations:** 1 VDF FutureCeuticals2692 N State Rt. 1-17, Momence, Illinois, 60954; 2 University of Illinois at Urbana-Champaign1201 W. Gregory Dr, Urbana, Illinois, 61801; 3 International Chemistry Testing258 Main St., Suite 202, Milford, Massachusetts, 01757; 4 VDF FutureCeuticals, Applied BioClinical Facility16259 Laguna Canyon Rd, Irvine, California, 92618

**Keywords:** DHR6G, fluorescence probe, free radicals, fruit and vegetable extracts, spectra

## Abstract

ORAC and other in vitro methods have to date proved useful in measuring antioxidant potential in foods. In order to better understand the potential relationship between diet and free radical production/mitigation, an in vivo analytic method can provide new insight into directly measuring reactive oxidant species (ROS). Dihydrorhodamine-6G (DHR6G) is indiscriminate to the various free radicals found in humans, and therefore can be useful in quantifying total ROS in vivo. Our aim was to investigate whether the total ROS in human subjects can be quantified using DHR6G after intake of a blend of antioxidants-rich fruit and vegetable-based materials. Twelve participants were given 100 mg of a proprietary blend of fruit, vegetable, and herb powders and concentrates commercially marketed under the trade name “Spectra™”. Blood samples were collected at 0, 60, 120 and 180 min and were subsequently tested for ROS in serum using DHR6G as a fluorescent probe. By quantifying this fluorescence, we were able to measure ROS concentrations in human blood. This method is both reliable and efficient for evaluating the efficacy of antioxidants against ROS in vivo. Our data indicate that eleven participants responded to the intake of Spectra™ by significant decreases of ROS concentrations.

## Introduction

Free radicals are consistently formed in the human body as by-products of aerobic metabolism, generally as reactive oxidant species (ROS). The most common ROS in vivo are superoxide (O_2_^•−^), hydroxyl radical (OH^•^), peroxyl radical (RO_2_^•^), nitric oxide (^•^NO), and peroxynitrite (ONOO^−^). Due to their indiscriminate attacks on lipids, proteins, nucleic acids, and carbohydrates, it has been reported that ROS may be associated with many degenerative conditions including the aging process (Halliwell 2006). In order to better understand the potential relationship between diet and free radical production and/or mitigation, an in vivo analytic method to directly measure ROS is very important. Although there are various existing methods to assess oxidative damage caused by ROS, such as measuring lipid peroxidation products and DNA adducts (Thérond et al. 2000), no method exists to directly measure ROS concentrations in vivo.

In our previous study, we first demonstrated the use of DHR6G as a fluorometric assay for ROS in vivo (Nemzer et al. 2013). The theory behind using DHR6G is that nonfluorescent DHR6G will emit fluorescence after being oxidized by ROS. The emitted fluorescence is directly proportional to the concentration of ROS. When applied to biological samples such as serum, DHR6G is oxidized by ROS and creates the highly fluorescent rhodamine 6G. Therefore, rhodamine 6G fluorescence can be used as an index to quantify the overall ROS in biological fluids.

Dietary antioxidants are widely believed to scavenge and/or inhibit the production of free radicals in the body. While numerous methods have been developed to evaluate total antioxidant capacity (TAC) of food samples, a direct connection between the antioxidant capacity of a food material and any resultant activity against ROS in vivo has been elusive (Prior et al. 2003).

In this study, we examined the in vivo antioxidant or antiradical activity of 100 mg of a proprietary combination of fruit, vegetable, and herb powders and extracts (Spectra™; VDF FutureCeuticals, Inc., Momence, IL) standardized to a minimum TAC as measured by a series of oxygen radical absorbance capacity (ORAC)-based assays collected under the name ORAC 5.0, including ORAC, HORAC, NORAC, SORAC, and SOAC. Spectra™ has the following phytochemical compounds per 100 mg serving size: quercetin 10.8 mg; catechins 10.3 mg; chlorogenic acids 6.7 mg; vitamin C 1.2 mg; anthocyanins 0.5 mg; glucosinolates 0.1 mg; lycopene 43 mcg; alliin 20 mcg; and allicin 10 mcg.

After oral administration of 100 mg of Spectra™, ROS in the collected serum samples were quantified by the DHR6G assay. Our results demonstrated that eleven subjects showed statistically significant decreases of ROS concentrations after intake of Spectra™.

## Materials and Methods

### Materials and reagents

*Spectra*^*™*^*ORAC 5.0 blend (VDF FutureCeuticals, Inc.)*: A combination of the following fruit, vegetable, and herb extracts and concentrates: broccoli powder and broccoli sprouts concentrate, onion extract, tomato concentrate, dried carrot, spinach, kale concentrate, brussel sprout concentrate, whole coffee fruit extract, acerola extract, camu camu fruit powder, acai berry concentrate, mangosteen fruit concentrate, green tea extract, apple extract, turmeric concentrate, garlic powder, basil concentrate, oregano powder, cinnamon concentrate, elderberry concentrate, black currant extract, blueberry extract, sweet cherry powder, blackberry powder, chokeberry powder, raspberry powder, bilberry extract. The ORAC5.0 assay measures antioxidant activities against hydroxyl, peroxyl, peroxynitrite, singlet oxygen, and superoxide anion. Spectra™ is standardized to a minimum 40,000 *μ*mol/L trolox equivalent per gram of ORAC5.0 assay.

*Chemicals:* 6-hydroxy-2,5,7,8-tetramethylchroman-2-carboxylic acid (Trolox) and dihydrorhodamine 123 (DHR-123) were obtained from Sigma-Aldrich (St. Louis, MO). 3-Morpholinosydnonimine, hydrochloride (SIN-1) was purchased from Toronto Research Chemicals (North York, Ontario, Canada). Hydroethidine (HE) fluorescent stain (5-ethyl-5,6-dihydro-6-phenyl-3,8-phenanthridinediamine) was purchased from Polysciences (Warrington, PA). Dihydrorhodamine 6G (DHR 6G) was obtained from Molecular Probes (Eugene, OR). Rhodamine 6G was purchased from Aldrich (Milwaukee, WI). All other reagents were of the highest commercial grade and used without further purification.

### Clinical protocol

This study was conducted according to the guidelines set forth in the Declaration of Helsinki. All procedures involving human subjects were approved by the Institutional Review Board at Vita Clinical S.A. Avenida Circunvalacion Norte #135, Guadalajara, JAL, Mexico 44270 (Study protocol ABC-NCI-12-12-ANTX-1). All study subjects were given a written consent in which they completed health-related questions prior to selection. All subjects were in good health and none were using any type of medication or supplement for 15 days prior to the study. Additional inclusion criteria included the following: fasting male and female, healthy subjects between ages 18 and 50, with a body mass index of 25–36, free from any chronic illnesses or serious health problems, no alcohol or drug use, no history of organ transplantation, no surgery within the last 12 months, and no regular consumption of coffee, green tea or other fruits (or juices). A total of 24 participants were selected and randomly divided into two groups, active (12 people) or placebo group (12 people). Each participant was given a single dose of Spectra™ (100 mg), or a matching 100 mg dose of oat bran used as a placebo. Peripheral venous blood samples were drawn from an antecubital vein and collected in either lithium-heparin (green top) BD Vacutainer® tubes or anticoagulant-free (dry tubes) (BD Vacutainerranklin Lakes, NJ). Before 100 mg Spectra™ was orally ingested, blood was collected at “time zero” (T0). Subsequently, blood was drawn at 1 h (T60), 2 h (T120), and 3 h (T180) following treatment, Subjects were fasted and resting throughout the study. Immediately after collection, dry tubes containing blood samples were allowed to clot for 30 min. Both serum and plasma were collected after centrifugation at 1200*g* for 30 min (Swing-out rotor; Beckman Coulter, Palo Alto, CA). Samples were aliquoted, snap frozen and kept at −70°C until use.

### Sample preparation for antioxidant measurements

The sample preparation was conducted following the previous protocol (Mullen et al. 2011). Approximately 20 mg of Spectra was extracted with 20 mL of ethanol/water (70:30 v/v) for 1 h at room temperature on an orbital shaker. After centrifugation at 2600*g*, the supernatant of the extract was subjected to the TAC assay. The TAC includes the determination of radical scavenging capacities against five free radicals, namely, peroxyl, hydroxyl, peroxynitrite, superoxide anions, and singlet oxygen radicals. All results were expressed as*μ*mol/L Trolox equivalent per gram (*μ*mol/L TE/g) and the TAC was the sum of the five individual results.

### Peroxyl radical scavenging capacity (ORAC assay)

The ORAC assay was measured according to a previous report by Ou et al. (2002) and Huang et al. (2002) with modification. The FL600 microplate fluorescence reader (Bio-Tek Instruments, Inc., Winooski, VT) was used with an excitation wavelength of 485 (20 nm) and an emission wavelength of 530 (25 nm). The action of 2,20-Azobis(2-amidinopropane) dihydrochloride (AAPH) was used to generate the peroxyl radical. Fluorescein (FL) was used as a fluorescent probe to indicate the extent of damage from its reaction with the peroxyl radical. The antioxidant effect was measured by comparing the fluorescence time/intensity area under the curve of the sample to that of a control with no antioxidant. Trolox was prepared as the standard solution. Fluorescence was measured every minute up to 35 min.

### Hydroxyl radical scavenging capacity (HORAC assay)

The assay was modified according to a report by Ou et al. (2002). FL was used as a fluorescent probe. The antioxidant effect was measured by comparing the fluorescence time/intensity area under the curve of the sample to that of a control with no antioxidant. Trolox was used as the standard for calibration.

### Peroxynitrite scavenging capacity (NORAC assay)

Peroxynitrite (ONOO^−^) scavenging values were determined by monitoring the oxidation of DHR-123 based on a protocol by Chung et al. (2001). A stock solution of DHR-123 (5 mmol/L) was prepared in dimethylformamide, purged with nitrogen and stored at −80°C. A working solution of DHR-123 (final concentration, fc, 5 *μ*mol/L) diluted from the stock solution was placed on ice in the dark before the experiment started. The reaction buffer consisting of 90 mmol/L sodium chloride, 50 mmol/L sodium phosphate (pH 7.4), and 5 mmol/L potassium chloride with 100 *μ*mol/L (fc) diethylenetriaminepentaacetic acid (DTPA) was purged with nitrogen and placed on ice before use. ONOO^−^ scavenging was measured in a fluorescence reader with an excitation wavelength of 485 (20 nm) and emission wavelength of 530 (25 nm). Five minutes after treating with or without SIN-1 (fc 10 *μ*mol/L) or authentic ONOO^−^ (fc 10 *μ*mol/L) in 0.3 N sodium hydroxide, the background and final fluorescent signals were measured. Oxidation of DHR-123 increased by decomposition of SIN-1 gradually, whereas authentic ONOO^−^ rapidly oxidized DHR-123 with its final fluorescent signal being stable over time.

### Superoxide anion scavenging assay (SORAC assay)

The SORAC assay was conducted following the previously described method by Zhang et al. (2009). HE was used to measure the O_2_^−^ scavenging capacity. The mixture of xanthine and xanthine oxidase was used to generate O_2_^−^ radicals. Nonfluorescent HE was oxidized by O_2_^−^ to form a species of unknown structure that emits fluorescence signal at 586 nm. Addition of superoxide dismutase (SOD) inhibits the HE oxidation.

### Singlet oxygen scavenging assay (SOAC assay)

The SOAC assay was modified based on the previously described method by Zhao et al. (2003). HE was used as a probe to measure singlet oxygen. The mixture of H_2_O_2_ and MoO_4_^2−^ was used to generate singlet oxygen. 40 *μ*mol/L solution of HE, 2.635 mmol/L Na_2_MoO_4_ and 13.125 mmol/L H_2_O_2_ working solutions were prepared in N, N-dimethylacetamide (DMA). HE solution (125 *μ*L) was added to a well followed by addition of 25 *μ*L of 2.635 mmol/L Na_2_MoO_4_ and 25 *μ*L of 13.125 mmol/L H_2_O_2_, respectively. Singlet oxygen scavenging was measured in a fluorescence reader with an excitation wavelength of 530 nm and an emission wavelength of 620 nm.

### Preparation of rhodamine 6G standard solution

A portion (5.15 mg) of rhodamine 6G was dissolved into a 4 mL phosphate buffer solution (PBS) buffer to make a final concentration of 2.688 mmol/L.

### Preparation of DHR6G solution

A portion (1.08 mg) of DHR6G was dissolved into 300 *μ*L DMSO to make a stock solution in 8.098 mmol/L. A working solution (202.22 *μ*mol/L) of DHR6G was made by adding 50 *μ*L DHR6G stock solution into 1.950 mL PBS.

### Serum preparation

Serum samples were thawed, vortexed, and centrifuged at 16,000*g* at 4°C for 3 min, 240 *μ*L of supernatant was deproteinized using 720 *μ*L of methanol. The mixture was vortexed for 30 sec, and then centrifuged at 16,000*g* for 5 min at 4°C.

### Fluorescence measurement

The supernatant was evaporated to dryness, and reconstituted in 60 *μ*L PBS. Forty microliter of the above prepared sample and 60 *μ*L of DHR6G working solution were mixed well in a 96-well reading plate, and the fluorescence intensity (excitation: 485 nm, emission 545 nm) was recorded for 24 h with an interval of 5 min.

### Statistical analysis

All statistical analyses were performed with the SigmaPlot 11.0 (Chicago, IL).*P* value was calculated using a one-way ANOVA with the Holm-Sidak method, and*P* < 0.05 was considered statistically significant.

## Results

### Total antioxidant capacity of spectra

The TAC of the Spectra™ product is summarized in Table1.

**Table 1:** Total antioxidant activity for Spectra™

Activity against individual radicals	Result (*μ*TE/g)
Activity against peroxyl radicals	10,698
Activity against hydroxyl radicals	15,113
Activity against peroxynitrite	1099
Activity against superoxide anion	13,370
Activity against singlet oxygen	4174
Total activity	44,454

### Oxidation of DHR6G in the presence of serum extract

**Figure 1:**
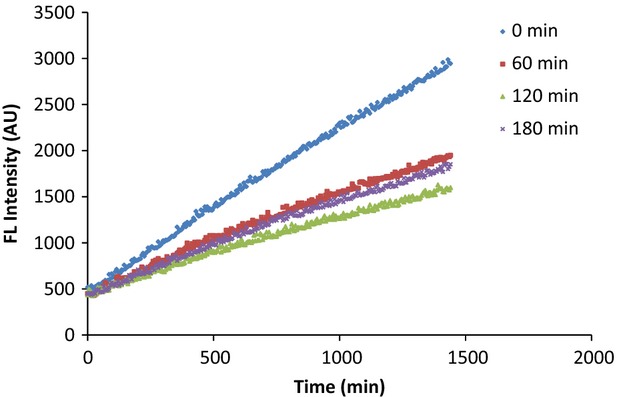
The changes in fluorescence intensity show that oxidation of DHR6G was significantly inhibited after intake of 100 mg Spectra (at 0 min). DHR6G, dihydrorhodamine-6G.

Originally, DHR6G was used in our previous study to detect and quantify smoke oxidants (Ou and Huang 2006). Through modification in the current study, DHR6G was able to be oxidized to rhodamine 6G during the course of incubation with the serum extract. Figure1 shows the measure of fluorescence intensity over the specified time period. It was observed that after 60 min of administration of 100 mg Spectra™, the rate of fluorescence intensity was significantly inhibited. This evidence suggests that Spectra™ was bioavailable in vivo and that the absorbed antioxidants neutralized endogenous free radicals.

### Stability of DHR6G to the air

**Figure 2:**
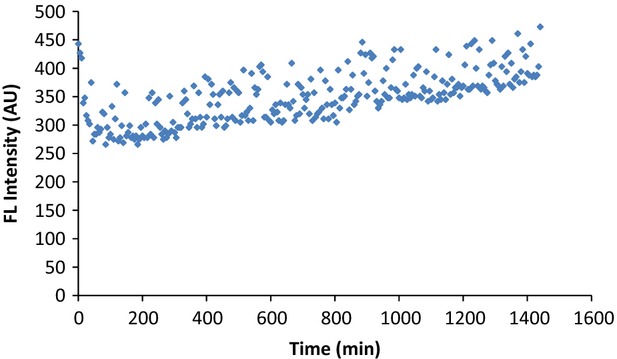
DHR6G at 0.406 *μ*mol/L was incubated with PBS buffer in a 96 plate well, and its fluorescence intensity was monitored for 24 h at 5-min intervals. Apparently, DHR6G was not auto-oxidized under these experimental conditions. DHR6G, dihydrorhodamine-6G.

**Figure 3:**
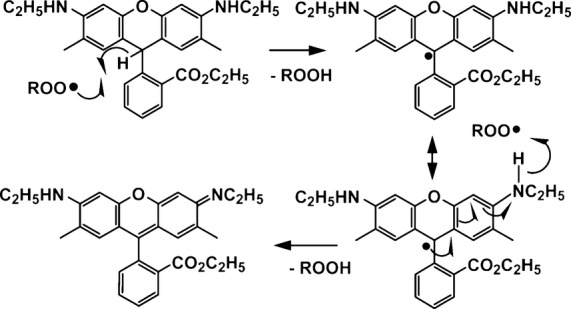
Mechanism of DHR6G oxidized by ROS. DHR6G, dihydrorhodamine-6G.

Due to the fact that DHR6G is labeled as “air-sensitive,” we tested its stability when exposed to the air by incubating it in PBS buffer. As shown in Figure2, during the 24-h period, only an insignificant change of fluorescence intensity was observed. On the contrary, in the presence of serum extract, the fluorescence intensity changes were apparent and linearly increased in a time sensitive manner (Fig.1). Therefore, we conclude that, under these experimental conditions, air has little contribution to any oxidation of DHR6G; instead, the ROS in the samples should be responsible for the formation of rhodamine 6G. From the reaction mechanism, one DHR6G molecule reacts with two radical molecules in order to form rhodamine 6G. This finding greatly simplifies the quantitation of ROS in vivo (Fig.3).

### Quantitation of the free radical concentrations

**Figure 4:**
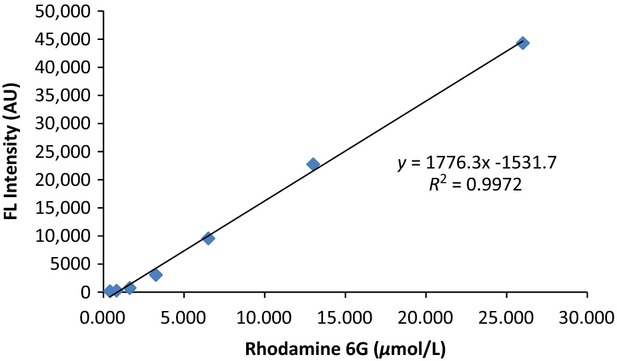
Standard curve of rhodamine 6G (0.406 to 26 *μ*mol/L).

The DHR6G reacts with the radicals present in prepared samples regardless of species. The concentration of rhodamine 6G formed in the reaction with free radicals is calculated from a standard curve obtained by plotting the known rhodamine 6G concentration versus the fluorescent intensity. The linearity ranges from 0.406 to 26 *μ*mol/L as shown in Figure4. Using this standard curve, the concentrations of rhodamine 6G formed during the reaction of free radicals with DHR-6G can be calculated. The stoichiometry between DHR-6G and free radicals is two. Thus,


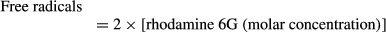


### Efficacy of Spectra™ in vivo

**Figure 5:**
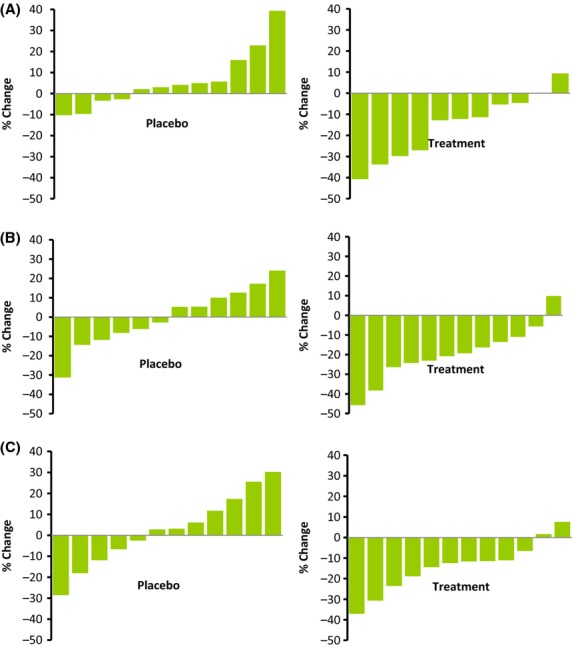
The change of serum-free radical concentrations in placebo and treatment groups at (A) 60, (B) 120, and (C) 180 min.

**Figure 6:**
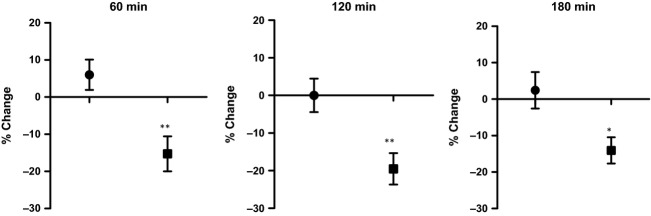
The change of serum-free radical concentrations in the placebo (•) and treatment groups (▪) at 60, 120 and 180 min. One asterisk (*) indicates significant difference at*P* < 0.05 71 compared with the placebo group by Student's*t* test; two asterisk (**) indicates significant difference at*P* < 0.005 compared with the placebo group by Student's*t* test.

The efficacy of Spectra™ is demonstrated in Figures5, 6. Figure5 shows that in the placebo group, for most participants, free radical concentrations in serum were not decreased, while in the Spectra™ group, as shown in Figure6, there was a significant decrease in serum-free radical concentrations across the entire group within three hours of consumption of 100 mg of Spectra™.

## Discussion

Accumulated evidence indicates that reactive oxygen species, such as peroxyl radicals (ROO·), hydroxyl radicals (HO·), the superoxide anion (O_2_^−·^), peroxynitrile (OONO^−^) and singlet oxygen (^1^O_2_), are involved in the pathophysiology of aging. To counteract the damage of the reactive oxygen species on living cells, the human body has a biological defense system that neutralizes the reactive oxygen species or prevents the reactive oxygen species from being generated in the first place. Depending on the reaction mechanisms, antioxidants are often classified into two major categories: radical chain-breaking antioxidants and preventive antioxidants. Chain-breaking antioxidants convert reactive free radicals (e.g., HO·) to stable and thus nonaggressive molecules through hydrogen atom transfer reactions between HO· and the antioxidants. As a result, the auto-oxidation chain reactions between the free radicals and the cellular molecules are terminated. Preventive antioxidants inhibit the oxidation reaction from occurring by either converting the precursors of the reactive oxygen species to unreactive species or inhibiting the oxidation reaction (Finkel and Holbrook 2000).

To counteract the assault of the superoxide anion reactive species, living cells have a biological defense system composed of enzymatic antioxidants that convert reactive oxygen species or reactive nitrogen species to harmless species. In contrast, no enzymatic action is known to scavenge ROO•, HO•, ^1^O_2_, and ONOO^−^, consequently the burden of defense relies on a variety of nonenzymatic antioxidants such as vitamin C, vitamin E, and many naturally occurring phytochemicals that have the property of scavenging oxidants and free radicals (Blacker et al. 2012).

To comprehensively evaluate the oxidant-scavenging capacity of a food sample, assays have to be designed to measure the activity of any given food stuff on reactive oxygen species. To date, however, the majority of commercially available assays are designed to measure a sample's capacity to react with one oxidant, the peroxyl radical (whether as an organic radical or a redox active metal complex). The peroxyl radical has been the most frequently used reactive oxygen species in antioxidant capacity assays because it is considered the most relevant radical in lipid auto-oxidation and can be generated conveniently from azo compounds. The peroxyl radical has been used, e.g., as a radical source in the widely used ORAC.

It is evident that the antioxidant defense “team” in living cells contains individual antioxidants with very different and specific tasks in the battle against oxidative stress and reactive oxygen species. Therefore, in order to comprehensively evaluate the antioxidant capacity of food nutrients in vitro, it was imperative that a broad range of assays that can cover all aspects of antioxidant capacity would be developed and validated. Although there is a validated assay for peroxyl radical absorbance capacity (ORAC), no such assay had been developed for any other of the reactive oxygen species until Ou et al. (2002) and Zhang et al. (2009) first published methods for the analysis of antioxidant capacity using the hydroxyl and superoxide anion radicals. Subsequently, we have addressed the other oxygen radical species by developing assays that assess their contribution to TAC. As shown in Table1, Spectra™ showed high TAC against various free radicals; in particular, the hydroxyl radical scavenging activity and the super oxide anion scavenging activity account for roughly 60% of the total free radical scavenging capacity of the Spectra™ formula. This is clearly indicative of the broader insight that might be gained by expanding our understanding and analyses of radical absorbance capacity beyond ORAC.

The most frequently applied methods for measuring free radicals use spin trap and electron spin resonance. These methods are also used to detect free radicals in vivo. Spin trapping coupled with ESR or HPLC is useful in that it directly detects free radicals in vivo (Quaresima and Ferrari 1998; Villamena and Zweier 2004). Indeed, the use of these techniques has been considered the gold standard method for free radical identification and quantitation. However, these techniques are largely inaccessible due to the high costs of the instrumentation.

Therefore, there is a need to develop an alternative method to affordably measure total oxidant species at a high throughput. The fluorescent probe used in the method described herein is sensitive to common ROS including peroxyl radicals, alkoxyl radicals, hydroxyl radicals, superoxide anion, peroxynitrite, and hypochlorous acid. Each of these ROS possesses strong oxidizing power and RHD-6G acts against each as a good reductant:


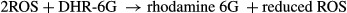


DHR6G is inexpensive and its oxidation can be easily monitored by a fluorescence plate reader, an instrument commonly found in most analytical labs. Although this method does not differentiate between individual free radical species, the protocol described within this study could be ideal for economical quantification of the total amount of ROS in vivo.

Spectra™ is an all-natural combination of fruits, vegetables and herbs rich in polyphenols, and possesses high antioxidant capacity as measured by its activity against five free radicals. Although the antioxidant capacity of polyphenols has been proposed as one of several potential in vivo mechanisms for maintenance of good health, evidence in human studies from this aspect has been limited. This is due to the lack of appropriate analytical methods for measuring the direct effect of antioxidant-rich materials against ROS in vivo. Current methodologies largely rely on measuring specific antioxidant metabolites by LC/MS; however, those metabolites are usually present at ultralow concentrations that make quantitation difficult. Additionally, they do not reflect the potential health benefits of intake of antioxidants.

Logically, in vivo antioxidant efficacy can be determined by examining the relationship between TAC of a given material and general in vivo ROS concentrations before and after ingestion of the same material. Development of a meaningful analytical method aimed at these two aspects has the potential to stimulate better research and product formulation. To date, we are unaware of any human studies reporting any direct impact of polyphenol-rich food materials on ROS concentrations. Accordingly, we report here the use of DHR6G as a novel approach to measuring this relationship using Spectra™ as an example. Our results showed that the plant-based antioxidants present in Spectra™ functioned well in vivo, and that the DHR6G assay can be a potentially valuable new tool for in vivo oxidative stress studies. We are in the process of continuing to investigate further possible correlations between TAC of food-based materials and their effects on total ROS concentrations.
